# The Role of Sensorimotor Processes in Pain Empathy

**DOI:** 10.1007/s10548-019-00738-4

**Published:** 2019-11-08

**Authors:** Igor Riečanský, Claus Lamm

**Affiliations:** 1 Social, Cognitive and Affective Neuroscience Unit, Department of Basic Psychological Research and Research Methods, Faculty of Psychology, University of Vienna, Liebiggasse 5, 1010 Vienna, Austria; 2 Department of Behavioural Neuroscience, Centre of Experimental Medicine, Institute of Normal and Pathological Physiology, Slovak Academy of Sciences, Sienkiewiczova 1, 813 71 Bratislava, Slovakia; 3 Cognitive Neuroscience, International School for Advanced Studies, Via Bonomea 265, 34136 Trieste, Italy

**Keywords:** Emotional contagion, Somatosensory system, Motor resonance, Mirror neurons, Nociception, Defensive system

## Abstract

Pain is a salient, aversive sensation which motivates avoidance, but also has a strong social signaling function. Numerous studies have shown that regions of the nervous system active in association with first-hand pain are also active in response to the pain of others. When witnessing somatic pain, such as seeing bodies in painful situations, significant activations occur not only in areas related to the processing of negative emotions, but also in neuronal structures engaged in somatosensation and the control of skeletal muscles. These empathy-related sensorimotor activations are selectively reviewed in this article, with a focus on studies using electrophysiological methods and paradigms investigating responses to somatic pain. Convergent evidence from these studies shows that these activations (1) occur at multiple levels of the nervous system, from the spinal cord up to the cerebral cortex, (2) are best conceptualized as activations of a defensive system, in line with the role of pain to protect body from injury, and (3) contribute to establishing a matching of psychological states between the sufferer and the observer, which ultimately supports empathic understanding and motivate prosocial action. Future research should thus focus on how these sensorimotor responses are related to higher-order empathic responses, including affective sharing and emotion regulation, and how this motivates approach-related prosocial behaviors aimed at alleviating the pain and suffering of others.

## Introduction

The ability to interact with the environment in an adequate way is fundamental for survival and requires a tight coupling between sensory and motor processes. In humans (and animals in general), multiple sensory channels provide the information about the environment and there are countless possibilities of different motor actions. Thus, to ensure safe and adaptive interaction with the environment, the modality specific sensory signals must be appropriately integrated to guide motor activity, whose consequences must in turn be monitored by additional or the same sensory systems, and so forth. For humans, from the very beginning of their lives, a substantial part of environmental interactions includes those with other human beings. It is thus not surprising that sensorimotor processes seem to be at the roots of social cognition (Legrand 2006; Farmer and Tsakiris 2012; Gallese 2014; Hari et al. 2015). One important social cognitive capacity is empathy, which enables us to share and experientially understand the feelings of other human beings (for review see e.g., Coplan and Goldie 2011). Converging evidence now shows that the neuronal mechanisms that are engaged in our emotional but also sensory and motor states are also engaged while observing other individuals being in those states (for recent reviews see e.g., Betti and Aglioti 2016; Coll and Jackson 2016; Zaki et al. 2016; de Waal and Preston 2017; Lamm et al. 2019). Research in pain empathy has made a particularly significant contribution to this discovery, benefiting from the fact that pain is not only a strong ubiquitous experience, but also a salient social signal. In addition to being an unpleasant sensory and emotional bodily experience, pain is also a strong driver of motor activities to avoid injury when interacting with the physical as well as social environment. It is thus not surprising that the sensorimotor activations related to first-hand noxious stimulation also occur in response to pain in others (e.g., Keysers et al. 2010; Lamm et al. 2011, for review). When facing an unexpected sudden and salient injury of another person we tend to painfully grimace, groan, carry out self-protective movements or touch the places on our bodies that correspond to other’s pain locations. In this review, we aim to provide a brief and selective overview of the role of basic somatosensory and somatomotor processes in pain empathy (Table 1). After providing a general background, we first focus on sensory and thereafter on motor processes, but often discussing them together due to their functional coupling at many levels of the neuraxis. Our review deliberately focuses on sensorimotor responses in areas of the brain considered as earlier or more “bottom-up” sensory-driven (in terms of the processing hierarchy), and their investigation using paradigms that assessed empathy using “flesh and bone” paradigms showing pain and injuries inflicted on the bodies of others. This choice was made based on limitations in space, but also since several recent reviews exhaustively covered other related topics (e.g., Betti and Aglioti 2016; Coll and Jackson 2016; Lamm et al. 2016; Coll et al. 2017; Kanske et al. 2017; Adriaense et al. 2019a). This implies that we will not review more complex empathic responses, including questions related to affect sharing or more “cognitive” aspects and the regulation of empathic responses, nor will we cover the large body of literature investigating empathy using (facial) emotion expressions or more abstract paradigms engaging mentalizing and perspective taking.

**Table 1:** Sensorimotor processes implicated in the processing of others’ pain

Somatosensation
Nociception
Bodily self-awareness
Motor excitability
Motor processing for protective movements

## The Neural Representation of First-Hand Pain

Pain is associated with neuronal activity in a set of brain regions often labeled together as pain matrix or neuromatrix, based on the concept of a functional network which internal interactions underlie the experience of pain (Melzack 1999; Iannetti and Mouraux 2010; Garcia-Larrea and Peyron 2013; Kucyi and Davis 2015). The main target regions of nociceptive afferents conveyed by the spinothalamic tract (Dum et al. 2009) are usually considered as the core of the pain matrix and include the thalamus, postcentral gyrus (primary somatosensory cortex, S1), parietal operculum (secondary somatosensory cortex, S2), the insula, and the dorsal-anterior/anterior-midcingulate cortex (dACC/aMCC) (Apkarian et al. 2005). The major elements of the pain matrix seem to be linked to different aspects of the multiple aspects of the pain experience. In particular, the activity in the lateral thalamus, postcentral gyrus and operculoinsular cortex, the so-called lateral nociceptive pathway, has been related to the sensory aspects of pain (such as location, character and intensity), while the activity in the medial thalamus, anterior insula and the dACC/aMCC (the medial nociceptive pathway) with the affective and motivational aspects (unpleasant emotional state, arousal, pain-related behaviors) (Schnitzler and Ploner 2000; Brooks and Tracey 2005; Auvray et al. 2010). Yet, the role of the pain matrix in pain experience remains debated since several recent findings suggest that the activity of the core regions of the pain matrix may be related to high intensity or salience rather than specific sensory quality (painfulness) of noxious stimuli (Mouraux et al. 2011; Salomons et al. 2016; for review see e.g. Iannetti et al. 2013; Mouraux and Iannetti 2018).

## Empathy for Pain: A Role for Both Affective and Sensorimotor Responses

In early functional neuroimaging studies, responses to pain in others were revealed mainly in the anterior insula and the dACC/aMCC, suggesting a predominant contribution of the medial nociceptive pathway and thus emotional processing to pain empathy (Singer et al. 2004; Morrison et al. 2004; Jackson et al. 2005; Botvinick et al. 2005). However, it soon became apparent that seeing pain inflicted on others also activates somatosensory and motor cortices (Avenanti et al. 2005; Bufalari et al. 2007; Lamm et al. 2007; Ogino et al. 2007). Such activations were detected in particular when participants in the studies were observing explicit depictions of somatic pain, such as when seeing injuries, wounds or body parts in painful situations, i.e. stimuli providing direct information about the sensory character as well as the bodily location of the nociceptive signals (Lamm et al. 2011). Activity in sensorimotor areas tends to be increased also when seeing facial expressions of pain, although these responses are less regular and seem to be weaker as those to body parts in painful situations (Saarela et al. 2007; Budell et al. 2010; Vachon-Presseau et al. 2012; Gallo et al. 2018). On the other hand, symbolic cues that are used in some experimental paradigms to indicate the presence of pain in others activate the sensorimotor brain areas to a much lower extent and with much higher variation across individuals and studies (Lamm et al. 2011).

A great part of evidence on the activation of the sensorimotor system has come from human electroencephalographic and magnetoencephalographic (EEG/MEG) studies. For instance, it was repeatedly confirmed that observing noxious, compared with innocuous, bodily events is accompanied by a suppression (so called “desynchronization”) of the Rolandic mu (7–12 Hz) and beta (13–30 Hz) EEG/MEG oscillatory rhythms (Cheng et al. 2008b, 2014; Perry et al. 2010; Whitmarsh et al. 2011; Chen et al. 2012; Riečanský et al. 2015, 2019; Hoenen et al. 2015; Motoyama et al. 2017; Fabi and Leuthold 2017; Levy et al. 2018), considered as a signature of cortical sensorimotor processing (Hari and Salmelin 1997; Crone et al. 1998; Pfurtscheller and da Silva 2005). Several studies reported that event-related modulation of the central mu and beta oscillations was associated with empathic abilities (Cheng et al. 2008a, b; Yang et al. 2009; Perry et al. 2010; Woodruff et al. 2011; Woodruff and Klein 2013; Riečanský et al. 2015). Furthermore, using MEG Betti et al. (2009) found that observation of painful hand stimulations increased coherence of gamma-band (> 30 Hz) oscillations between somatosensory and motor cortex. Electrophysiological methods may thus be more sensitive than functional magnetic resonance imaging (fMRI) to detect sensorimotor activations. Alternatively, these differences between methodologies might relate to a wider use of bodily depictions in EEG/MEG studies on pain empathy.

## Zooming in on the Role of Somatosensation in Empathy for Pain

However, the brain sources of the scalp-recorded electromagnetic signals (in particular EEG) cannot be assessed with sufficiently high spatial precision to reliably disentangle activations in closely adjacent cortical regions, such as the posterior vs. the anterior bank of the central sulcus (see also Arnstein et al. 2011). This is a major shortcoming as these areas may differ in their functional specialization within the nociceptive or the sensorimotor system. Research using fMRI, which has higher spatial resolution, has reliably revealed though that observing noxious bodily stimulation elicits activation of primary and secondary somatosensory cortex (Lamm et al. 2007, 2011; Keysers et al. 2010). The somatosensory cortex responds significantly also when observing innocuous bodily contacts, i.e. touch of other bodies (Keysers et al. 2004; Blakemore et al. 2005; Schaefer et al. 2009, 2012; Gazzola et al. 2012; Kuehn et al. 2014), and this activation positively correlates with trait empathy (Kuehn et al. 2013). Furthermore, several studies have shown that observing pain in others modulates somatosensory evoked potentials elicited by non-painful stimuli, which are generated in the somatosensory cortex (Bufalari et al. 2007; Pihko et al. 2010; Voisin et al. 2011; Martínez-Jauand et al. 2012; Canizales et al. 2013; Marcoux et al. 2013). In line with these findings, it has been reported that observing pain in others enhances tactile perception (Morrison et al. 2013b; Vandenbroucke et al. 2014a, b). Interestingly, subjects with mirror-touch or mirror-pain synesthesia, who experience tactile sensations or pain when they see someone else being touched or painfully injured, show increased activation of the somatosensory cortex to observed touch or pain compared with non-synesthetes (Blakemore et al. 2005; Osborn and Derbyshire 2010). Furthermore, somatosensory cortex is also activated by observing other’s actions (Gazzola and Keysers 2009) and in situations when painful consequences of interactions with noxious objects are expected (Morrison et al. 2013b). These findings offer a broader functional perspective of the significance of the “empathic” somatosensory activations, which are addressed in the following paragraphs.

## Bodily Self-Awareness, and Protection of Peripersonal Space as Possible Mechanisms in Responding to Others’ Pain?

Among the targets of somatosensory inputs are posterior parietal and premotor cortical areas, which provide an integrated multisensory-motor representation of body and space immediately surrounding body (peripersonal space, PPS). There is now firm evidence that these representations play a crucial role in bodily interactions, body protection and in the awareness of self (Graziano and Cooke 2006; Brozzoli et al. 2014; di Pellegrino and Làdavas 2015; Bufacchi and Iannetti 2018; Serino 2019). The study by Costantini et al. (2008) has shown that premotor and parietal areas are also activated by observing noxious stimulations of hands, indicating their contribution to pain empathy. Furthermore, it has been shown that manipulations of bodily self-awareness, such as inducing of bodily illusions, exert influence on first-hand pain (Hänsel et al. 2011; Höfle et al. 2012; Romano et al. 2014, 2016), but also affect the processing of social information (Maister et al. 2015b; Maister and Tsakiris 2016). Remarkably, susceptibility to bodily illusions is positively associated with trait empathy (Asai et al. 2011; Farmer et al. 2012; Seiryte and Rusconi 2015). We have recently tested whether bodily illusions modulate neural responses to pain of others and found that inducing illusory self-attribution of another’s hand increased suppression of the central mu and beta EEG oscillations during observation of pain inflicted on the another’s hand so that participants with stronger illusion showed stronger empathy-like activations (Riečanský et al. 2019). These results suggest a link between pain empathy and bodily self-awareness, which may be achieved by flexible extensions of PPS to include other individuals into the body protective zone of the observer (Maister et al. 2015a; Teramoto 2018; Serino 2019). This interpretation is supported by the fact that PPS is plastic and undergoes adaptive shifts with changing possibilities of bodily interactions with objects and other humans (Martel et al. 2016; Serino 2019).

## How Pain in Others Influences the Processing and Perception of Nociceptive Stimuli

Perhaps not surprisingly based on the information in previous paragraphs, in addition to interfering with the processing of tactile stimuli, exposure to pain of others also affects nociception in the observer. As mentioned, the experience of pain is both sensory and emotional and both these aspects seem to be affected by observing pain in others. For instance, Loggia et al. (2008) reported that observing an actor receiving painful heat stimuli increased both unpleasantness and intensity of pain elicited by prolonged heat stimuli, which indicates that both the emotional and the sensory dimensions of pain were modulated by other’s pain. Moreover, the effects on pain perception in the observers were stronger if their attitudes toward the actor were positive. Similarly, observing body parts in painful situations increased both unpleasantness and intensity of pain elicited by electric pulses (Vachon-Presseau et al. 2011). These observations thus indicate that seeing pain in others might affect activity in both the medial and the lateral nociceptive pathways in response to first-hand noxious stimuli. However, Godinho with co-workers reported that an increase in the intensity of pain to noxious electric stimuli, that occurred when observing bodily injuries, was accompanied by modulation of late components of somatosensory evoked potentials and the activity in the prefrontal cortex, pregenual cingulate cortex, and temporo-parietal-occipital junction, but not in the somatosensory cortex or other core areas of the pain matrix (Godinho et al. 2006, 2012).^1^ Interestingly, in contrast to pain elicited by electric pulses or sustained heat, pain sensations evoked by brief laser heat stimuli were not changed during observation of hands undergoing needle penetrations in two studies (Valeriani et al. 2008; Valentini et al. 2012). It is thus possible that perception of brief heat pain is not affected by seeing pain in others. Alternatively, the modulations of first-hand pain may be modality specific and occur only in case the noxious stimuli delivered to the other and the self are of the same physical modality, which remains to be empirically tested. Furthermore, Valeriani et al. (2008) reported that viewing noxious body stimulation specifically affected early components of laser evoked potentials (LEPs) to stimuli directed to the hand: LEPs were reduced during observation of needle penetrations into hands, but not feet or when observing innocuous contacts of hand. Hence, the effects on LEPs only occurred if the model and the participant were painfully stimulated at the same body location. These results thus differ from those of Godinho and co-workers (Godinho et al. 2006), although the two studies differed in the type of visual stimuli (pictures of injuries vs. videos of hands in first-person perspective), noxious stimulation (electric vs. laser),^2^ but also participants’ instructions (Valeriani et al.: to imagine the displayed hand was their own). Moreover, the modulation of LEPs was not replicated in a study by Valentini et al. (2012), who rather found an effect on beta-band EEG oscillations, which was interpreted as a marker of motor inhibition. Note also that the fMRI study by Krishnan et al. (2016) indicates that somatotopic patterns of the somatosensory cortical activations for first-hand pain and imaging pain in oneself (as a means to induce empathy) are not identical.

To sum up, although several investigations have found that observing pain in others alters the processing of the sensory quality of noxious stimuli experienced in oneself, there is little agreement among the studies about the precise neural mechanisms of these interactions. This may be related to limited knowledge on the neuronal mechanisms of pain, as a multimodal experience going beyond nociception. This argument can be illustrated e.g. by findings from patients with congenital insensitivity to pain. These patients are unable for nociception in response to noxious stimuli, due to a genetic deficit (Bennett and Woods 2014). However, they have learned to classify stimuli as painful, probably due to associative learning but possibly also based on activating higher-order and possibly domain-general aspects of pain processing. This may explain why these patients, when exposed to pain in others, show brain activation as well as pain ratings largely comparable (but not identical) to healthy subjects (Danziger et al. 2009). However, these patients underestimate pain in others when they are only shown stimuli of body parts in painful situations, i.e. without the associated (e.g. facial or vocal) pain expressions—although neural responses of their somatosensory cortex to such stimuli are preserved (Danziger et al. 2006). Furthermore, despite having no nociceptive sensations, and a (lack of a) subjective experience of pain that starkly deviates from healthy controls, these patients show normal activation of the “pain matrix” when exposed to first-hand noxious stimuli, which are perceived as painful by healthy individuals (Salomons et al. 2016).

These findings seem to indicate that sharing or at least understanding the pain of others is possible in the absence of first-hand nociception. This is important for an ongoing debate centered on the question whether empathy for pain indeed relies on a specific engagement of the neural processes engaged in the first-hand representation of pain (Corradi-Dell’Acqua et al. 2011, 2016; Hayes and Northoff 2012; Rütgen et al. 2015a, b, 2018; Krishnan et al. 2016; for recent reviews see e.g., Zaki et al. 2016; Lamm et al. 2016, 2019). While this debate has predominantly focused on shared representations regarding the affective-motivational rather than the sensory-discriminative component of pain, it nevertheless seems of relevance for the present review as well. More specifically, it opens the question whether empathy-related activations of sensorimotor processes indeed indicate a specific sharing of the other person’s pain, in one’s own sensorimotor pain processing system. Due to the correlative nature of the experimental designs and the neuroscientific research methods used in most of previous research, answering this question might require novel approaches enabling causal manipulations and thus conclusions on the specific role of somatosensory and motor responses in triggering affective empathy-related responses (see e.g., Gallo et al. 2018). In this, the currently available repertoire of human neuroscience methods is naturally limited, which is why there seems great potential in comparative (but mostly invasive) research, which has recently gained considerable traction both in classical as well as alternative model species, such as rodents and ravens, respectively (Chen 2018; Carrillo et al. 2019; Adriaense et al. 2019b, c). This notwithstanding, our own group is currently extending the psychopharmacological approach previously used to demonstrate affect sharing to more specifically investigate the role of somatosensory processes in placebo empathy analgesia—with preliminary data suggesting a rather unspecific role of somatosensation (Hartmann et al. 2018, 2019). This should not imply though that somatosensory processes do not play a role in empathy, but rather that their role might be other than a specific sharing of the somatosensory aspects of pain as well.

## Spinal Nociception and Protective Reflexes

There is evidence that viewing noxious stimulations modulates nociception at the level of spinal cord (Vachon-Presseau et al. 2011; Mailhot et al. 2012). This is indicated by a facilitation of the nociceptive flexion reflex (NFR), an automatic withdrawal reaction of a limb to a painful stimulus which is mediated by spinal circuitry (Sandrini et al. 2005; Morrison et al. 2013a). The facilitation of NFR is not selective for observing pain though and occurs when exposed to aversive stimuli not depicting pain as well, indicating a general role of this reaction in avoidance of threat. An fMRI study by Roy et al. (2009) has shown that the enhancement of NFR with negative primes is associated with the activity in several brain regions including pons, thalamus, amygdala and medial prefrontal cortex, highlighting that multiple mechanisms at different levels may contribute to sensitization of spinal nociception. Importantly, observing pain in conspecifics leads to NFR facilitation also in rats, allowing for direct investigation of the cellular and molecular mechanisms of the “empathic” enhancement of spinal nociception (Chen 2018). Li et al. (2014) have reported that the increase in NFR following pain observation is associated with an enhanced activity of spinal dorsal horn neurons. In line with the role of the ACC in the control of spinal nociception (Xiao and Zhang 2018), lesions of the ACC and the adjacent medial prefrontal cortex prevented the “empathic” facilitation of the NFR (Li et al. 2014). In agreement with these findings, deactivation of ACC in rats reduces freezing while witnessing foot shocks to another rat (Carrillo et al. 2019). Furthermore, recent experiments suggest that the “empathic” enhancement of the NFR is mediated by the locus coeruleus-noradrenergic system and the peripheral P2X3 nociceptor (Lü et al. 2017). This finding is interesting given the existence of a direct projection from the ACC to the locus coeruleus, which is assumed to play an important role in behavioral adaptations to pain as well as other salient experiences (Aston-Jones and Cohen 2005). It is generally acknowledged that ACC plays a pivotal role in the transformation of pain and other negative emotions into aversively-motivated behavior including avoidance and escape (Vogt 2005; Shackman et al. 2011; Heilbronner and Hayden 2016; Xiao and Zhang 2018). The studies reviewed above indicate that ACC triggers activation of defensive motor programs also in response to pain in others. This fits well with empathy-related responses in the midbrain periaqueductal gray matter and cerebellum (Jackson et al. 2005; Cheng et al. 2007; Lamm and Decety 2008; Lamm et al. 2011; Gu et al. 2012; Braboszcz et al. 2017), areas involved in nociception as well as defensive behaviors (Moulton et al. 2010; Linnman et al. 2012; Coombes and Misra 2016; Koutsikou et al. 2017).

## Empathy for Pain and Skeletomotor Activity

A considerable number of pain empathy studies investigated the activity of the skeletomotor system by means of transcranial magnetic stimulation (TMS). Motor corticospinal excitability (CSE) can be assessed by applying single magnetic pulses over the primary motor cortex and measuring the amplitude of the elicited motor evoked potentials (MEPs) recorded from the corresponding skeletal muscle. In a series of studies, Avenanti et al. have shown that observing needle penetration into a muscle, compared with observing touch at the same location, resulted in a decreased MEPs from the corresponding muscle of the observer (Avenanti et al. 2005, 2006, 2009a, b; Minio-Paluello et al. 2006). This modulation of CSE was muscle specific, thus MEPs of the first dorsal interosseus muscle (FDI) of the observer’s right hand were reduced only in case they observed penetration of the FDI of the right hand. Furthermore, the effect depended on the bodily intervention (present for penetration, absent for pinprick), ethnic group membership (present for ingroup, absent for outgroup hands), and correlated with the intensity of pain attributed to the model, as well as with self-reported empathic traits (see also Fecteau et al. 2008). The authors also demonstrated that CSE of the corresponding muscle of the opposite hand is elevated rather than inhibited. A parallel has been drawn to an inhibitory effect of first-hand muscular pain on the excitability of motor cortex (Nijs et al. 2012). However, arguments have been raised that the modulations of CSE reflect the situational context so that the inhibition of CSE is the consequence of the fact that the painful stimulation cannot be avoided (De Coster et al. 2014). Furthermore, Bucchioni et al. (2016) demonstrated that the inhibition of CSE occurs only when seeing the other’s hand in the first-person perspective, but not in the third-person perspective, and argued that the CSE inhibition may reflect an illusory bodily ownership (“embodiment”) of the other’s hand (also see Fossataro et al. 2018 and de Guzman et al. 2016). Using the same stimuli as Avenanti et al., our own group has documented that seeing needle penetrations, compared with seeing touch, elicits stronger suppression of sensorimotor mu and beta EEG oscillations, and that this effects gets stronger with increasing illusory ownership of the observed hand (Riečanský et al. 2015, 2019). These findings indicate that sensorimotor cortex is activated rather than inhibited by seeing needle penetrations into the hand from the first-person perspective and that the activation increases with increasing embodiment (Evans and Blanke 2013; Takemi et al. 2013). These results apparently contradict the findings from the TMS studies and the reasons for these differences across methodologies are currently not clear. In pain synaesthetes, for instance, observing hands in painful scenarios from the egocentric perspective elicits elevation, not reduction, of CSE (Fitzgibbon et al. 2012a) and a stronger suppression of central mu and beta EEG rhythms compared to control subjects (Fitzgibbon et al. 2012b; Grice-Jackson et al. 2017). Furthermore, Morrison et al. demonstrated that observing a needle pricking a fingertip facilitates withdrawal and inhibits approach movements (Morrison et al. 2007b). Several studies also indicate that observing pain in others elicits a non-specific movement facilitation (Morrison et al. 2007a; Han et al. 2017; Galang et al. 2017; Fabi and Leuthold 2017). In particular, a recent study by Galang and Obhi (2019) has shown that observing needle penetrations into right hand from the egocentric perspective decreased latencies of button presses to go signals carried out with the right hand and this response facilitation was stronger after explicitly instructing observers to empathize with a person in pain.

In sum, the effects of pain observation on the skeletomotor system are variable and depend on stimuli, viewing perspective, task, and task instructions. Combined TMS-EEG or TMS-fMRI experiments could help to reconcile the contradictory results from different methods. It should be also considered that the activity of the skeletomotor system in response to observed pain may be highly dynamic and may differ at various levels of the nervous system, as indicated by the effects of experimentally induced pain on CSE (Bank et al. 2013; Algoet et al. 2018).

## Summary and Conclusions

The selective literature review of this paper demonstrates that witnessing somatic pain in others triggers activation of sensorimotor processes in the observer. They seem at least in part automatic and largely driven by bottom-up sensory processes. In terms of the levels of processing hierarchies, they go down as low as at the level of the spinal cord—demonstrating the complexity and integrative nature of information processing in the central nervous system in order to provide an optimal link between sensory inputs to motor outputs. Our review furthermore suggests that sensorimotor activations to other’s pain seem best conceptualized as reflecting the activation of defensive responses in agreement with the goal of pain, which is to protect the body from external harm (Baliki and Apkarian 2015). They are also related to the role of pain in body representation and self-awareness. The activations seem mirror-like (also termed resonant), i.e. similar to those when the observers are themselves exposed to noxious stimuli (see however, Lamm and Majdandžić 2015, for a discussion of the role of mirror neurons in empathy; and Hartmann et al. 2019). It seems thus highly plausible that the activations of the sensorimotor system contribute to a basic experiential understanding of the sensorimotor, but also the affective experience and motivational state of others (Prochazkova and Kret 2017). While we deliberately did not focus on these “higher-order” aspects, they are probably the ones that have the strongest behavioral consequences of empathy and of empathic concern, i.e. prosocial behaviors such as comforting or helping another in pain (Bird and Viding 2014; Decety et al. 2016; de Waal and Preston 2017; Heyes 2018; Mafessoni and Lachmann 2019). In order to understand the complex architecture of empathy, it thus remains a major challenge for future research in social neuroscience to address the relationship between the “low-level” aversion-motivational state, promoting defensive self-oriented actions, and a “high-level” approach-motivational state, underlying consolation and active helping behavior directed at the person suffering from pain, or other aversive states (Adriaense et al. submitted; Weisz and Zaki 2018; but see Bloom 2017). In Table 2 we provide a short list of open questions worth to be addressed in future studies in order to get deeper understanding of the nature and the role of sensorimotor processes in pain empathy.

**Table 2:** Selected open questions related to the role of sensorimotor processes in pain empathy

How are sensorimotor activations to others’ pain linked with other-related affective responses (empathic concern) and prosocial behaviors (consolation, helping)?
How do sensory responses to others’ pain differ from those to others’ touch?
How does pain in others act on nociceptive pain experienced by oneself?
Can pain empathy be up- or down-regulated by the modulation of sensorimotor processes, e.g. with pharmacological agents or neurostimulation?

## Footnotes

1 This is similar to studies investigating event-related potentials (ERPs) to visual stimuli of pain in others, which show modulation of late (“cognitive”, “affective”) rather than early (“sensory”) ERP components (for review, see Coll 2018).

2 Note that although laser stimuli, in contrast to electric pulses, selectively activate afferent nociceptive fibers, LEPs do not reflect nociceptive-specific neural activity (Mouraux and Iannetti 2009).
